# New perspective to evaluate N1 staging: The peripheral lymph node metastasis status of non‐small cell lung cancer

**DOI:** 10.1111/1759-7714.13213

**Published:** 2019-10-16

**Authors:** Jiaqi Zhang, Lei Liu, Guige Wang, Cheng Huang, Yeye Chen, Ye Zhang, Chao Guo, Shanqing Li

**Affiliations:** ^1^ Department of Thoracic Surgery Peking Union Medical College Hospital, Chinese Academy of Medical Sciences and Peking Union Medical College Beijing China

**Keywords:** Lymph node metastasis, N stage, non‐small cell lung cancer, peripheral lymph node

## Abstract

**Background:**

Lymph node (LN) metastasis status is the decision‐making basis for the surgical procedure and adjuvant therapy modalities. Fewer studies have previously focused on LN metastasis in N1 station, especially on peripheral lymph node (PLN) metastasis in N1 station. This study aimed to reveal the metastasis status of PLN of non‐small cell lung cancer (NSCLC), and investigate its effects on N staging.

**Methods:**

We retrospectively evaluated a consecutive series of patients who underwent curative resection for histologically confirmed N1 NSCLC. Propensity score matching (PSM) was used to analyze the effects of PLN on N staging.

**Results:**

A total of 105 patients with confirmed pathological N1 (pN1) stage NSCLC with solitary nodule and without neoadjuvant therapy were enrolled into the study: 55 patients had intraperipheral LN metastasis (IPLNM), and 50 patients had extra‐peripheral LN metastasis (EPLNM). Before PSM analysis, type of location (*P* = 0.002), surgical procedure (*P* = 0.008), number of positive LNs (*P* = 0.029), number of LNs removed (*P* = 0.010), lobe of lung cancer (*P* = 0.031), and vascular invasion (*P* = 0.049) showed significant differences between the two groups. After PSM analysis, statistically there were differences in type of location (*P* = 0.034), number of positive LNs (*P* = 0.008) and vascular invasion (*P* = 0.049) between them.

**Conclusion:**

PLN metastasis was a quite common pattern of LN metastasis in N1 station of NSCLC. IPLNM occurred more frequently in central NSCLC and NSCLC with vascular invasion, and thoracotomy was likely to secure more accurate PLN staging. Clinicians should pay great attention to PLN dissection. Follow‐up data will be needed in order to detect the prognosis of IPLNM patients.

## Key points

### Significant findings of the study

We found that patients with N1 station lymph node metastasis accounted for nearly 33.60% of patients with LN metastasis. Peripheral lymph node metastasis was quite a common pattern of LN metastasis in N1 station.

### What this study adds

Peripheral lymph nodes appear to play a more important role in N staging.

## Introduction

Primary lung cancer has become the leading cause of cancer‐related death around the world, with non‐small cell lung cancer (NSCLC) accounting for about 85% of lung cancer cases. The five‐year survival rate of lung cancer patients has decreased significantly with the upregulation of NSCLC stage, according to some reports, and the five‐year survival rate of patients with stage IA NSCLC may be more than 70%, while that of patients with stage III is significantly lower than 40%, and that of patients with stage IV is no more than 10%.^1^ Owing to the successful screening for lung cancer, most lung cancer patients can be diagnosed and treated early. At present, for NSCLC patients, especially those diagnosed as clinical stage I, II and IIIa, surgical treatment still forms the basis of comprehensive treatment. LN metastasis status is one of the key factors affecting the prognosis of patients with NSCLC, and is related to therapeutic strategy.

Regional LNs of lung cancer metastasis was first proposed by Naruke *et al*. in the 1970s.^2^ The International Association for the Study of Lung Cancer (IASLC) synthesized Naruke *et al*. and MD‐ATS maps^3^ in 2009 to redefine the anatomic definitions of lung cancer LN stations.^4^ LN metastasis of lung cancer is usually divided into three stations, namely N1/N2/N3 station LNs, closely related to the tumor‐node‐metastasis (TNM) staging of lung cancer, consequently predicting the prognosis of patients.

According to the latest edition, lung cancer TNM stage classification, N1 station LNs are divided into two zones, including hilar/interlobar zone (regrouping levels 10 and 11) and peripheral zone (regrouping levels 12, 13, 14), which is proposed for future survival analysis.^5^ Previously, many studies have focused on LN metastasis in N2 station,^6^, ^7^, ^8^ but fewer studies have focused on LN metastasis in N1 station, especially on PLNs. This study aimed to reveal the PLN metastasis status of NSCLC, and investigate its effects on N staging.

According to the influences of PLN on N staging, N1 station LN metastasis was classified into two subtypes: intraperipheral LN metastasis (IPLNM) and extra‐peripheral LN metastasis (EPLNM). The IPLNM group were patients with NSCLC with PLN metastasis only (including single or multiple levels of 12, 13 and 14), and did not have LN metastasis in the hilar/interlobar zone (level 10 and 11). The EPLNM group were patients with NSCLC who showed LN metastasis in the hilar/interlobar zone (level 10 and 11), with/without PLN metastasis. All patients with NSCLC did not have N2 station LN metastasis.

## Methods

### Patients

We retrospectively evaluated a consecutive series of patients who had undergone curative resection for histologically confirmed pN1 NSCLC between January 2017 and May 2019 at the Peking Union Medical College Hospital (PUMCH). This study was reviewed and approved by the Institutional Review Board of PUMCH.

All patients clinical staging evaluation was confirmed preoperatively using positron emission tomography/computed tomography (PET/CT), or using chest and abdomen computed tomography combined with enhanced brain magnetic resonance imaging/CT and whole‐body bone scintigraphy. The eighth edition lung cancer stage classification was used for staging. The selected patients were divided into the IPLNM and EPLNM groups.

Patients included met the following criteria: (i) They had received the first surgical intervention at our clinical center; (ii) the surgical technique was lobectomy and systematic LN dissection and (iii) post‐surgery pathological examination confirmed NSCLC, and existence of N1 station LN metastasis.

**Figure 1:**
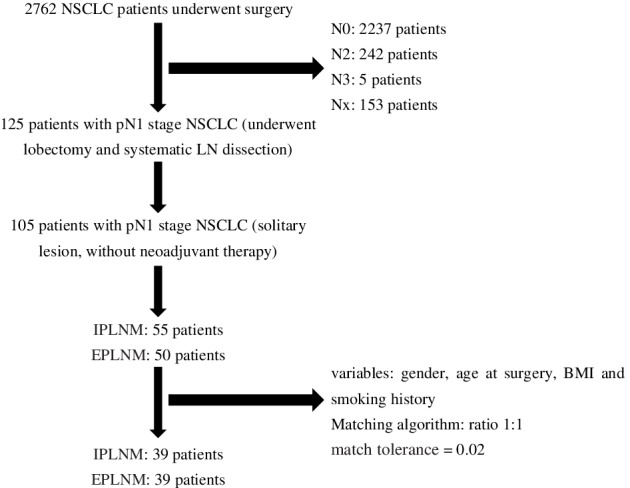
Flowchart showing patient enrolment and propensity score matching process

Our exclusion criteria included the following: (i) neoadjuvant therapy; (ii) multiple lung cancer; (iii) lung cancer invading the chest wall, pericardium, phrenic nerve, mediastinum, diaphragm, heart, great vessel, recurrent laryngeal nerve, carina, trachea, esophagus, spine; (iv) malignant pleural/pericardial nodules or extra‐thoracic metastasis; (v) N2/N3 station LN metastasis and (vi) positive surgical margins (R1 or R2 resection) (Fig 1). Patients classified pathologically as carcinoid, mucoepidermoid carcinoma, or adenoid cystic carcinoma were also excluded.

### Surgical technique

The surgical procedures were conducted by senior thoracic surgeons at PUMCH according to the same oncological and surgery principles. After surgical and oncological evaluation, R0 resection by anatomic pulmonary resection and systematic mediastinal LN dissection was performed. The LN map was used as a reference in the eighth edition of TNM classification of lung cancer issued by the IASLC.^4^ During surgery, the senior thoracic surgeon classified the resected LNs into different levels according to the map; we classified levels 12, 13 and 14 together, grouped by PLN. All specimens were then sent for pathological examination.

### Data collection

The clinicopathologic variables of the patients were reviewed and included the following: gender, age at surgery, body mass index (BMI), smoking history, type of location (central or peripheral lung cancer), maximum diameter of tumor, lobe of lung cancer, obstructive pneumonia visible on imaging, treatment with antibiotics before surgery, surgical procedure (video‐assisted thoracic surgery [VATS] or thoracotomy), visceral pleural invasion, bronchus invasion, vascular invasion, spread through air spaces (STAS), number of positive LNs, number of LNs removed, and pathological histologic subtype.

### Propensity score matching analysis

To minimize selection bias between the two groups, a propensity score matching (PSM) analysis was performed. The ratio of patients in each group was 1:1, the match tolerance was therefore set at 0.02, and the following variables used for the PSM analysis: gender, age at surgery, BMI and smoking history.

### Statistical analysis

All statistical analyses were performed with IBM SPSS statistics, version 23.0 (International Business Machines Corp., Armonk, New York, USA). The chi‐squared test or Fisher's exact test was used for bivariate two‐sided comparisons of categorical variables between the groups. *P* < 0.05 (two‐sided) was considered significant.

## Results

### Patient characteristics

A total of 2762 patients who received surgery were pathologically confirmed with NSCLC, among whom 2237 patients showed no LN metastasis, and 125 patients had N1 station LN metastasis; 242 patients had N2 station LN metastasis,five had N3 station LN metastasis, and 153 could not be accessed because the number of LNs removed was not enough for staging. Patients with N1 station LN metastasis accounted for nearly 33.60% of patients with LN metastasis. If PLNs had not been dissected and carefully sorted, 55 cases of N1 cases would have been misdiagnosed as N0. Without examination of the PLNs, 55 of the 2292 cases would have been misdiagnosed as pN0 and the false‐negative rate for N staging would be 2.40%.

Overall, 105 patients with confirmed pN1 NSCLC, who underwent curative resection between January 2017 and May 2019, met the eligibility criteria and were selected for this study analysis, of whom 55 were placed into the IPLNM group, and 50 into the EPLNM group.

### Prematch study population

Baseline characteristics of the prematch groups are shown in Table 1. There were statistically significant differences between the two groups in type of location (*P* = 0.002), surgical procedure (*P* = 0.008), number of positive LNs (*P* = 0.029), number of LNs removed (*P* = 0.010), lobe of lung cancer (*P* = 0.031), and vascular invasion (*P* = 0.049).

**Table 1:** Demographic, clinical and pathological characteristics of patients before and after propensity score matching

	Prematch			Post‐match		
Variables	EPLNM group	IPLNM group	*P*‐value	EPLNM group	IPLNM group	*P*‐value
Number	50	55		39	39	
Age (year)	58.74 ± 10.13	61.42 ± 9.24	0.160	61.62 ± 8.94	60.05 ± 9.83	0.465
Gender (case %)			0.563			0.500
Female	22 (44.00)	24 (43.64)		15 (38.46)	16 (41.03)	
Male	28 (56.00)	31 (56.36)		24 (61.54)	23 (58.97)	
BMI (kg/m^2^)	24.35 ± 2.74	23.64 ± 3.32	0.234	24.19 ± 2.96	23.52 ± 3.32	0.350
Smoking history			0.541			0.500
No	25 (50.00)	27 (49.09)		17 (43.59)	18 (46.15)	
Yes	25 (50.00)	28 (50.91)		22 (56.41)	21 (53.85)	
Maximum diameter of tumor	2.85 ± 1.30	2.99 ± 1.19	0.554	2.87 ± 1.35	2.82 ± 1.134	0.870
Type of location (case %)			0.002			0.034
Central	6 (12.00)	21 (38.18)		6 (15.38)	14 (35.90)	
Peripheral	44 (88.00)	34 (61.82)		33 (84.62)	25 (64.10)	
N status in imaging (case %)			0.403			0.500
No	39 (78.00)	45 (81.82)		31 (79.49)	32 (82.05)	
Yes	11 (22.00)	10 (18.18)		8 (20.51)	7 (17.95)	
Obstructive pneumonia (case %)			0.567			0.215
No	45 (90.00)	50 (90.91)		34 (87.18)	37 (94.87)	
Yes	5 (10.00)	5 (9.09)		5 (12.82)	2 (5.13)	
Treatment with antibiotics (case %)			0.185			0.606
No	41 (82.00)	40 (72.73)		30 (76.92)	30 (76.92)	
Yes	9 (18.00)	15 (27.27)		9 (23.08)	9 (23.08)	
Surgical procedure (case %)			0.008			0.080
VATS	45 (90.00)	38 (69.09)		34 (87.18)	28 (71.79)	
Thoracotomy	5 (10.00)	17 (30.91)		5 (12.82)	11 (28.21)	
Number of positive LNs	2.28 ± 1.34	1.76 ± 1.04	0.029	2.38 ± 1.39	1.64 ± 0.99	0.008
Number of LNs removed	19.56 ± 9.68	25.25 ± 12.34	0.010	19.79 ± 10.07	23.21 ± 11.30	0.163
Visceral pleural invasion (case %)			0.496			0.320
No	29 (58.00)	33 (60.00)		23 (58.97)	26 (66.67)	
Yes	21 (42.00)	22 (40.00)		16 (41.03)	13 (33.33)	
Bronchus invasion (case %)			0.164			0.240
No	21 (42.00)	17 (30.91)		16 (41.03)	12 (30.77)	
Yes	29 (58.00)	38 (60.09)		23 (58.97)	27 (69.23)	
STAS (case %)			0.295			0.500
No	46 (92.00)	53 (96.36)		37 (94.87)	38 (97.44)	
Yes	4 (8.00)	2 (3.64)		2 (5.13)	1 (2.56)	
Vascular invasion (case %)			0.049			0.049
No	34 (78.00)	46 (83.64)		27 (69.23)	34 (87.18)	
Yes	16 (32.00)	9 (16.36)		12 (30.77)	5 (12.82)	
Lobe of lung cancer (case %)			0.031			0.159
Left upper lobe	7 (14.00)	19 (34.55)		7 (17.95)	14 (35.90)	
Left lower lobe	20 (40.00)	9 (16.36)		13 (33.33)	5 (12.82)	
Right upper lobe	10 (20.00)	13 (23.64)		7 (17.95)	10 (25.64)	
Right middle lobe	2 (4.00)	3 (5.45)		2 (5.13)	2 (5.13)	
Right lower lobe	11 (22.00)	9 (16.36)		10 (25.64)	7 (17.95)	
Left whole lung	0 (0.00)	2 (3.64)		0 (0.00)	1 (2.56)	
Pathological histologic subtype (case %)			0.086			0.209
Adenocarcinoma	38 (76.00)	32 (58.18)		29 (74.36)	25 (64.10)	
Squamous carcinoma	7 (14.00)	19 (34.55)		6 (15.38)	13 (33.33)	
Adenosquamous carcinoma	2 (4.00)	2 (3.64)		1 (2.56)	0 (0.00)	
Mucinous adenocarcinoma	1 (2.00)	2 (3.64)		1 (2.56)	1 (2.56)	
Large cell carcinoma	2 (4.00)	0 (0.00)		2 (5.13)	0 (0.00)	

### Post‐match baseline characteristics

Details of the PSM process are shown in Figure 1. Demographics and clinicopathological features of all 78 of these matched patients (*n* = 39 for each group) are shown in Table 1. The two groups differed significantly in type of location (*P* = 0.034), number of positive LNs (*P* = 0.008) and vascular invasion (*P* = 0.049). Central lung cancer was larger than peripheral lung cancer (3.4074 ± 1.36521 vs. 2.7590 ± 1.15943; *P* = 0.033).

Moreover, consistent with the results before PSM, there was no difference in obstructive pneumonia, or treatment with antibiotics before surgery after PSM between the two groups.

## Discussion

Anatomic pulmonary resection is a standard procedure for patients with NSCLC, and sublobar resection and lung‐sparing anatomic resection is preferable for some patients. Lymphatic metastasis is the major and most common route of metastasis for NSCLC. LN dissection is not only used for curative resection of potential resident tumor cells, but also for accurate staging, which makes LN dissection an important part of curative therapy of lung cancer. N1 and N2 station LN resection should be conducted for curative resection, even for patients who have undergone sublobar resection, and the appropriate N1 and N2 LN stations sampled unless not technically feasible. The minimum requirement for LNs which should be assessed pathologically is that at least six LNs should be removed; three from N1 and three from N2 stations.^9^ The scope of systematic LN dissection includes mediastinal LN levels 2R, 4R, seven, eight, and nine for right side lung cancer, and mediastinal LN levels 4L, five, six, seven, eight, and nine for left side lung cancer. Both sides should have N1 station LN levels 10, 11 and 12 resected.^10^

Studies have revealed that LN metastasis status is significantly associated with local recurrence and overall survival.^11^ Previous studies have focused on N2 station LN metastasis due to its poor prognosis and complicated therapy pattern. Some studies have revealed that the subclassification of N1 LNs (single N1 involvement and multiple N1 involvement) were associated with the prognosis of patients with NSCLC.^12^, ^13^ However, fewer studies have focused on the zone of N1 LN metastasis, and there is little knowledge about N1 station LN dissection, especially with regard to the dissection scope of N1 station LNs. At present, most surgeons do not proceed further than N1 station LN resection and classification at the levels 10, 11 and 12, and hardly any clinical attention is given to resection and sorting of intrapulmonary LNs, especially PLN. Preoperative CT does not appear to be a reliable method to evaluate LN metastasis, particularly intrapulmonary LN metastasis.^14^

Naruke *et al*. was the first to report in detail the LN metastasis status of different levels in N1 station. The results indicated that a sentinel node for a small sized tumor, irrespective of the location of tumor, was level 12, 11, and/or 10 in N1 station, and that dissection or sampling are a prerequisite for staging.^15^ However, this study showed that PLN metastasis was a common pattern of LN metastasis in N1 station. Most patients confirmed as pN1 stage showed IPLNM, instead of LN metastasis in the hilar or interlobar zone.

Currently, one of the indications for segmentectomy is without hilar or mediastinal LN metastasis proven by fast‐frozen pathology during surgery. However, the results of this study showed that most of the patients with N1 station LN metastasis had PLN metastasis, although the average diameter of lung cancer in this study was more than 2 cm. If PLN was not resected or carefully evaluated, 2.4% of patients might be downstaged to pN0. Xiao *et al*. conducted a prospective study and the results showed that it was possible for lung cancer to metastasize to the unaffected segment within the same lobe.^16^ Therefore, the resection and sorting of N1 station LNs in lung cancer is crucial. Besides mediastinal and hilar LN metastasis, PLN metastasis should be taken into consideration when segmentectomy is conducted. This conclusion provides a novel perspective to evaluate whether segmentectomy would be safe. We should be alert to the LN metastasis in N1 station. However, because the tumor was relatively large in this study, which is generally not indicated for segmentectomy or wedge resection, so we expect more data about PLN to confirm the safety of sublobar resection.

The results indicate that type of location and vascular invasion differed significantly between the two groups. The tumor size of central lung cancer was larger than peripheral lung cancer (3.41 ± 1.37 vs. 2.76 ± 1.16; *P* = 0.033), which perhaps explains why IPLNM occurred more frequently in the central one. Thoracotomy seems to be a better option to detect PLN metastasis, but the selected patients for thoracotomy might have several difficulties for VATS. Benign lesion such as obstructive pneumonia and treatment with antibiotics before surgery do not seem to be related to N staging.

In addition, studies have shown that the greater the number of LN metastases, the worse the prognosis in both pN1 and pN2 diseases.^13^ Demir *et al*. reviewed 540 patients with pN1 NSCLC, and reported that patients with hilar LN involvement had lower survival rates than patients with peripheral N1.^17^ In this study, the number of LNs in IPLNM metastasis was less than that of EPLNM, but patients with IPLNM were more likely to have vascular invasion. Whether there is a difference in prognosis between the two groups remains to be confirmed.

There are several limitations to our study. First, we did not follow‐up these patients owing to our enrolled patients recently undergoing surgery. Second, although the PSM process can reduce potential biases in retrospective studies, unlike randomized controlled trials, the biases caused by unobserved variables cannot be eliminated. To our knowledge, this is the first study focusing on the IPLNM status of NSCLC. Therefore, we hope that in the future there will be further research, particularly in the form of large, randomized prospective studies, which focus on this topic to further verify our results.

In conclusion, this study demonstrated that PLN metastasis was a fairly common pattern of LN metastasis in N1 station, which made it an important part of N staging. Central lung cancer was also more likely to metastasize to PLN. Open surgery might be more beneficial to improve the accuracy of N1 staging, and vascular invasion was more common in IPLNM. Attention should be paid to the adequate resection and detection of LNs in N1 station. We are also looking forward to the results of further studies into the survival rate differences in the different groups.

## Disclosure

The authors declare there are no conflicts of interest.
